# The Role of Fatty Acid Amide Hydrolase, a Key Regulatory Endocannabinoid Enzyme, in Domain-Specific Cognitive Performance in Psychosis

**DOI:** 10.1093/schbul/sbae212

**Published:** 2024-12-27

**Authors:** Ana Weidenauer, Ranjini Garani, Nittha Lalang, Jeremy Watts, Martin Lepage, Pablo M Rusjan, Romina Mizrahi

**Affiliations:** Division of General Psychiatry, Department of Psychiatry and Psychotherapy, Medical University of Vienna, Vienna 1090, Austria; Comprehensive Center for Clinical Neurosciences and Mental Health, Medical University of Vienna, Vienna 1090, Austria; Clinical and Translational Sciences Lab, Douglas Research Centre, Montreal, Quebec H4H 1R3, Canada; Integrated Program in Neuroscience, McGill University, Montreal, Quebec H3A 1A1, Canada; Vertex Pharmaceuticals, Boston, MA 02210, United States; Research Centre, CHU Sainte-Justine, Montreal, Quebec H3T 1C5, Canada; Department of Psychiatry, Université de Montréal, Montreal, Quebec H3T 1J4, Canada; Douglas Research Centre, Montreal, Quebec H4H 1R3, Canada; Department of Psychiatry, McGill University, Montreal, Quebec H3A 1A1, Canada; Integrated Program in Neuroscience, McGill University, Montreal, Quebec H3A 1A1, Canada; Douglas Research Centre, Montreal, Quebec H4H 1R3, Canada; Department of Psychiatry, McGill University, Montreal, Quebec H3A 1A1, Canada; Clinical and Translational Sciences Lab, Douglas Research Centre, Montreal, Quebec H4H 1R3, Canada; Integrated Program in Neuroscience, McGill University, Montreal, Quebec H3A 1A1, Canada; Douglas Research Centre, Montreal, Quebec H4H 1R3, Canada; Department of Psychiatry, McGill University, Montreal, Quebec H3A 1A1, Canada

**Keywords:** [^11^C]CURB, positron emission tomography, first-episode psychosis, clinical high risk for psychosis, cognition

## Abstract

**Background and Hypothesis:**

Cognitive impairments are particularly disabling for patients with a psychotic disorder and often persist despite optimization of antipsychotic treatment. Thus, motivating an extension of the research focus on the endocannabinoid system. The aim of this study was to evaluate group differences in brain fatty acid amid hydrolase (FAAH), an endocannabinoid enzyme between first-episode psychosis (FEP), individuals with clinical high risk (CHR) for psychosis and healthy controls (HCs). Furthermore, to test the hypothesis that FAAH is linked with cognition using positron emission tomography (PET).

**Study Design:**

We analyzed 80 PET scans with the highly selective FAAH radioligand [^11^C]CURB, including 30 patients with FEP (6 female), 15 CHR (5 female), and 35 HC (19 female). The Repeatable Battery for the Assessment of Neuropsychological Status (RBANS) and the Berg Card Sorting Test (BCST) were applied to test cognitive performance.

**Study Results:**

There was no difference in FAAH activity between groups (*F*_2, 75_ = 0.75, *P* = .48; Cohen’s *f* = 0.141; small effect). Overall, there was a difference in the association between groups regarding FAAH activity and the domain visuospatial construction (*F*_2, 72_ = 4.67, *P* = .01; Cohen’s *f* = .36; medium effect). Furthermore, across the sample, lower FAAH activity was associated with a higher percentage of perseverative responses (*F*_1, 66_ = 5.06, *P* = .03; Cohen’s *f* = 0.28, medium effect).

**Conclusions:**

We report evidence for associations between endocannabinoid alterations in FEP and CHR with specific domains of cognition (visuospatial construction and perseverative response), not overall cognition.

## Introduction

Schizophrenia is a psychiatric disorder with a frequently occurring severe course^1^ particularly for cognitive impairment, despite adequate antipsychotic treatment of positive symptoms.^2^ Cognitive impairment remains one of the most disabling features of psychosis^3^ and poses an exceptional challenge for treatment as well as for the individual’s everyday life.^4^ Although dopamine-based biomarkers have been associated with cognitive impairment in schizophrenia,^5–7^ dopamine receptor D_2/3_ antagonists often do not alleviate or even aggravate these symptoms^8^ necessitating the investigation of other neurotransmitter systems.

Here, we investigate, for the first time, the role of the endocannabinoid system (eCB) in cognition in these populations as the eCB regulates neuronal signaling in the human brain^9–13^ and shows alterations at various levels in psychosis.^14^ One of the most abundant endogenous cannabinoids in the human brain, *N*arachidonoylethanolamide, anandamide, acts mostly on cannabinoid 1 (CB1) receptors^15^ and is catabolized primarily by fatty acid amide hydrolase (FAAH^16^). Initial positron emission tomography (PET) studies investigating CB1 receptors in the brain of patients with schizophrenia demonstrated increased binding,^17,18^ while recent studies showed lower CB1 receptor levels in the brain of patients with psychosis.^18–21^ Furthermore, increased levels of anandamide in patients with schizophrenia and CHR in comparison to healthy controls (HCs) have been measured in peripheral blood and cerebrospinal fluid.^22–25^ Activity of FAAH in the human brain on the other hand, as measured with PET and the radioligand [^11^C]CURB, was not altered in patients with early psychosis as compared to controls. However, lower FAAH activity predicted higher positive symptom severity in patients.^26^

It seems legitimate to suspect a crucial role of endocannabinoids in cognitive processes in psychotic disorders. While the cognition-impairing effects of delta-9-tetrahydrocannabinol (THC) are well established, and for cannabidiol there might be no cognition-altering effects^27^ or slightly pro-cognitive effects in patients with schizophrenia^28^ and healthy volunteers,^29^ the role for endogenous cannabinoid markers including FAAH on cognitive markers remains unknown. Using peripheral assays it has been shown that lower FAAH^30^ and increased CB2 expression were associated with worse cognitive performance,^31^ while in the brain reduced CB1 receptor availability has been associated with lower performance in patients with psychosis^32^ and better visuospatial performance in healthy groups.^33^ Curiously, some work hints to pro-cognitive effects of endocannabinoid signaling. A study in Alzheimer’s patients showed a positive association between cognitive parameters and the participants’ postmortem brain anandamide levels^34^ while studies using FAAH inhibitors showed no enhancement of cognitive performance in healthy subjects.^35^ Similar ambiguity emerges when one examines animal studies, which showed no effects of FAAH inhibitors on cognition^36–38^; while others showed cognition-promoting effects.^39–44(p2)^

Given the previous findings that lower FAAH activity is associated with higher positive symptom severity,^26^ investigating its role in cognitive processes could reveal novel neuroscientific targets. While previous studies have produced mixed results regarding the cognitive effects of FAAH modulation, our study aims to clarify the role of FAAH in well-defined clinical populations. To our knowledge, no study so far has examined FAAH in clinical high risk (CHR) for psychosis or investigated the relationship between cognition and FAAH activity in the living human brain neither in healthy adults, CHR, or FEP. Here, we leveraged our large PET dataset,^26^ including improvements to methods to increase statistical power. This study aims to investigate differences in FAAH activity across groups, including FEP, CHR, and HC, with a focus on the novel inclusion of CHR, which has not been studied in this context. Our primary objective is to determine whether FAAH levels differ among these groups, building on earlier findings that reported no significant differences in FAAH activity in early psychosis.^26^

Next, we will examine how FAAH activity is associated with cognitive performance across these groups. Specifically, we hypothesize that lower brain FAAH activity will predict worse cognitive performance, particularly in cognitive domains such as attention and executive functioning, typically affected in schizophrenia. Given the region-specific differences in FAAH, our exploratory analysis will also investigate variations across regions of interest (ROIs).

## Methods

### Participants

The study was performed under a repository protocol that allowed a re-analysis of previously acquired data approved by the Centre for Addiction and Mental Health Research Ethics Board and now approved under Clinical and Translational Sciences (CaTS) BioBank by the Research Ethics Board (REB) of the Centre intégré universitaire de santé et de services sociaux (CIUSSS) de l’Ouest-de-l’Île-de-Montréal—Mental Health and Neuroscience subcommittee for secondary analyses. All participants were able to comprehend the study to provide consent (established using the MacArthur Competence Assessment Tool for Clinical Research (MacCAT) on patients with first-episode psychosis (FEP) and CHR) in accordance with the Declaration of Helsinki. All participants were in the age range of 18–40 years old and screened negative for drugs of abuse and did not meet the criteria for a substance use disorder at the time of the study. Self-reported sporadic cannabis use in the last 12 months and lifetime use was allowed and carefully examined during an interview and the amount of grams (g) was estimated for both time periods as exactly as possible.^26^ HCs were not included if they or first-degree family members met the criteria for any psychotic disorders. For the CHR group, participants with a diagnosis CHR using Structured Interview for Psychosis Risk Syndromes (SIPS^45^), classified “moderately ill” on the Clinical Global Impression scale, with no current DSM-IV-TR axis I diagnosis at the time of the study were included. First-episode psychosis had to fulfill DSM-IV criteria for either schizophrenia, schizoaffective disorder, schizophreniform disorder, delusional disorder, or psychosis not otherwise specified. Moreover, the onset of manifest psychotic symptoms, duration of illness, and duration of untreated psychosis were assessed during a thorough clinical interview and a review of clinical notes, whenever available. Treatment history of FEP and CHR was carefully recorded and antipsychotic medication was converted into chlorpromazine equivalents.^46^ We further excluded participants with a positive cannabis urine test and individuals with a manifest cannabis use disorder. Furthermore, subjects were not included if pregnant, breastfeeding, or if they had an unstable medical or neurological illness, a positive history of head trauma or if any contraindications for magnetic resonance imaging were present.

All data were collected between April 2013 and March 2020 (see Supplementary Material for CONSORT diagram). Individuals from this cohort partially overlap with study samples from previously published work (Watts et al., 2020).^45^ All participants with available cognition data from Watts et al. (2020)^45^ (*n* = 47; FEP = 25, HC = 22) were included in the present study. The previous study^45^ included a total of 63 participants in total, including 27 individuals with a psychotic disorder and 36 HCs. In our expanded cohort, we included an additional 33 participants (HC = 13, CHR = 15, FEP = 5), while excluding those without cognition data (*n* = 16; HC = 14 and FEP = 2), bringing the total number to 80 participants. Specifically, the current study includes 35 HCs (*n* = 19 female), 15 individuals with CHR for psychosis (*n* = 5 female), and 30 patients with FEP (*n* = 6 female) for whom RBANS data were available. However, Berg Card Sorting Test (BCST) was not available for 2 FEP participants, 1 CHR participant, and 2 HCs.

### Assessment of Cognitive Functions

The Repeatable Battery for the Assessment for Neuropsychological Status is a frequently used cognitive testing battery^47^ validated for patients with schizophrenia^48^ and was performed by trained research group members. In addition to a total score, the RBANS measures 5 cognitive domains: immediate memory, visuospatial construction, language, attention, and delayed memory. The RBANS index scores were derived using the normative data provided by the RBANS manual.^47^ We utilized the age-corrected normative values to calculate the index scores for our analyses.

To test executive functioning, a computerized open-source version of the BCST (http://pebl.sourceforge.net/)^49^ from the Psychology Experiment Building language (PEBL)^50^ was administered. The Wisconsin Card Sorting Test (WCST), from which this version has been derived, has been validated in patients with schizophrenia.^51^ The BCST has previously been applied in patients with schizophrenia.^52^ During the BCST, 64 cards are used and participants have to determine the rule by which cards have to be sorted (color, number, or shape) and are required to press 1 of 4 numbered keys based on the provided feedback (correct or incorrect). The rules change after a certain time and the participants have to adapt to the new rules. From the many scores that can be derived from the BCST output, we chose to analyze the total percentage of errors and the percentage of perseverative responses, as perseveration is a known phenomenon in schizophrenia.

### Assessment of Symptoms

The Positive and Negative Syndrome Scale (PANSS) was utilized in this study to evaluate the severity of symptoms in FEP participants.^53^ Among the various methods available to interpret PANSS results, we employed the 5-factor model, which categorizes symptoms into 5 distinct domains: positive symptoms (such as hallucinations and delusions), negative symptoms (including social withdrawal and lack of motivation), cognitive symptoms (impairments in thinking and processing information), depressive symptoms (feelings of sadness and hopelessness), and excitatory symptoms (such as hostility and impulsiveness).^54^ This 5-factor model approach provides a structured way to capture the complexity of symptom presentations in individuals experiencing psychosis.^54^ The Scale of Prodromal Symptoms (SOPS) was used to assess symptom severity in the CHR group. This scale evaluates 4 key symptom domains: positive, negative, disorganized, and general symptoms, providing a structured understanding of symptoms in individuals at risk for psychosis.^55^

### Positron Emission Tomography Using [^11^C]CURB

High-resolution PET was performed on a CPS/Siemens HRRT scanner (CPS/Siemens, Knoxville, TN) measuring radioactivity in 207 slices with a 1.2 mm interslice distance. After the transmission scan, [^11^C]CURB was injected over the course of 60 s (Harvard Apparatus, Holliston, MA) and data were acquired as previously described.^26^ For anatomic delineation of ROIs, a brain magnetic resonance image was acquired for each participant.^26^ Regions of interest were chosen based on involvement in cognitive processes^56^ and high FAAH content^26(p2)^: dorsolateral prefrontal cortex, medial prefrontal cortex, temporal cortex, anterior cingulate cortex, hippocampus, associative striatum, limbic striatum, sensorimotor striatum, and cerebellum. We quantified [^11^C]CURB with the validated 2-tissue-compartment model with irreversible binding compartment and arterial input function with 60 min data using image analysis and kinetic modeling pipelines (PELI v.2021.1 and FMOD v.1.7.3) that were developed and validated by Dr. Rusjan.^57,58^

Since the effect of FAAH (rs324420) genotype on [^11^C]CURB binding is well known, this polymorphism was determined for every subject as described previously^59^ and was entered as a covariate in statistical analyses. Participants were excluded if genotype was not available (*n* = 1).

### Statistical Analyses

For statistical analysis and for plot generation, R software (version 4.2.2^60^) and the packages ggplot2, rstatix, and lme4 were used. To evaluate differences in demographics between groups, *t*-tests and chi-square tests were applied. A check for outliers was performed by using the interquartile range (IQR) method. One healthy subject with poor BCST performance was an extreme outlier in both subtests as defined by being more extreme than Q1–3 * IQR or Q3 + 3 * IQR and was excluded from the BCST analysis. This subject had no relevant cannabis history and no such low performance on the RBANS test, which is why only the BCST data were removed from the analysis. Results including this outlier are shown in the Supplementary Material.

Linear mixed models were applied first to test differences in FAAH between study groups (FEP, CHR, and HC) and then to study the association between test scores and [^11^C]CURB λk_3_ values between study groups. For each domain, we first ran a model that included the interaction between study groups (FEP, CHR, and HC) and cognitive task performance. The models were specified to include fixed effects for group, task performance, and interaction between group and task performance controlling for FAAH rs324420 genotype, regions of interest, with subject ID specified as random intercepts. Analysis including common sources of confounds such as sex, age, years of education, smoking status, and previous cannabis exposure (past year and lifetime in g) as covariates were also tested (Supplementary Material). Covariates and interactions (group × score × ROI) were excluded from the model if proved nonsignificant. In order to replicate the previously published finding of the positive association between positive symptoms and FAAH activity in psychosis,^26^ we investigated the [^11^C]CURB γk3 and PANSS positive symptom subscale in the FEP group correcting for sex, age, FAAH *rs324420* genotype, and ROI. Similarly, we sought to explore the relationship between [^11^C]CURB γk3 and positive sub-SOPS in the CHR group.

## Results

### Demographics and Clinical Characteristics

A total of 80 [^11^C] CURB PET participants were analyzed, of which 35 were HCs, 15 CHR, and 30 FEP. This dataset partially overlaps with a previously published dataset (*n* = 49^26^). The demographics and clinical characteristics of the participants are presented in Table 1. There was a significant difference in sex distribution across groups with more male individuals in CHR and FEP. Also, FEP had a slightly higher age than the other 2 groups. Furthermore, the FEP cohort had significantly more individuals with previous cannabis experience and higher tobacco use. In the FEP group, 22 patients had diagnosis of schizophrenia, 5 had diagnosis of schizophreniform disorder, the 3 remaining subjects were diagnosed with delusional disorder, psychosis not otherwise specified, and schizoaffective disorder, respectively. As published in our previous work,^26^ there was a positive relationship between PANSS positive symptoms and FAAH activity in the FEP group, however, not reaching significance (*F*_1,26_ = 3.14, *P* = .088, controlling for ROI effect: *F*_8, 232_ = 65.65, *P* < .0001; *FAAH* rs324420 genotype effect: *F*_2, 26_ = 11.70, *P* = .0002). The result did not change when correcting for age, sex, or past year cannabis use. Significance, however, improved to a trend when including antipsychotic dose as a covariate (*F*_1, 25_ = 3.67, *P* = .067). There was no relationship with other symptom domains. A similar analysis was performed for the SOPS positive domain in the CHR group and no association was observed also without a change when covariates were added.

**Table 1.** Demographics and Characteristics of the Participants

Variable		HC (*n* = 35)	CHR (*n* = 15)	FEP (*n* = 30)	*F*/χ^2^	*P* value
Age (years), mean ± SD		22.99 ± 3.76	21.79 ± 2.13	25.12 ± 5.56	3.52	*P* = .03
Sex, female/male, *n*		19/16	5/10	6/24	8.24	*P* = .02
*FAAH* rs324420 genotype, CC/AC/AA, *n*		22/11/2	10/5/0	15/14/1	1.29	*P* = .87
PET parameters, mean ± SD	Molar activity (mCi/µmol)	1,896.16 ± 851.07	1,833.81 ± 1,143.81	2,136.24 ± 1,394.89	0.50	*P* = .61
	Mass injected (µg)	1.92 ± 0.85	2.02 ± 0.92	1.99 ± 1.32	0.05	*P* = .95
	Activity injected (mCi)	9.74 ± 0.68	9.38 ± 0.75	9.49 ± 0.81	1.60	*P* = .21
Antipsychotic (AP)-naïve/AP-free/AP-current, *n*		–	11/3/1	9/10/11	8.22	*P* = .01
AP equivalent dose of those medicated		–	34.44	264.36 ± 395.18	2.33	*P* = .10
Cannabis lifetime exposure (cannabis past users only, g)		4 ± 2.83	8.33 ± 20.54	610.1 ± 626.66	*5.13*	*P = .008*
Cigarettes per day (in smokers), mean ± SD		–	2.68 ± 2.50	8.63 ± 9.41	*1.12*	*P = .32*
Years education		15.27 ± 1.78	14.47 ± 1.77	14.47 ± 2.24	*2.6*	*P = .11*
RBANS scores	Immediate memory	96.06 ± 17.31	95.73 ± 13.99	89.63 ± 17.49	*1.33*	*P* = .27 *P*_FDR_ = 0.67^a^
	Visuospatial construction	83.31 ± 17.23	86.2 ± 15.79	86.07 ± 14.96	*0.30*	*P* = .74 *P*_FDR_ = 0.77^a^
	Language	86.74 ± 19.33	87.53 ± 16.04	91.33 ± 18.26	*0.44*	*P* = .65 *p*_FDR_ = 0.77^a^
	Attention	104.17 ± 16.25	93.67 ± 21.13	92.33 ± 14.96	*4.56*	*P *= .01 *P*_FDR_ = 0.06^a^
	Delayed memory	91.94 ± 13.51	92.67 ± 15.57	89.80 ± 14.79	*0.27*	*P* = .77 *P*_FDR_ = 0.77^a^
	Total score	89.89 ± 14.46	88.6 ± 18.18	87.00 ± 14.64	*0.29*	*P* = .75 *P*_FDR_ = 0.77^a^
BCST scores^*^	Errors %	12.03 ± 3.72	12.02 ± 4.45	12.90 ± 3.83	0.73	*P* = .40 *P*_FDR_ = 0.40^a^
	Perseverative responses %	28.83 ± 3.60	29.51 ± 3.85	30.79 ± 4.07	3.94	*P* = .05 *P*_FDR_ = 0.10^a^

^*^Results here are shown without BCST outlier.

^a^Significance for cognitive subtests was set at *P* less than .05; for false discovery rate (FDR) adjusted *P* values (*P*_FDR_), significance was set at *P*_FDR_ less than .10. *P* values were derived from ANOVA analyses.

Abbreviations: SD—standard deviation; HC—healthy control; CHR—clinical high risk; FEP—psychosis group Χ^2^—chi-squared statistic; *F*-value—ANOVA test statistic; AP—antipsychotic; mCi—millicurie; µmol—micromole; µg—micrograms; RBANS—Repeatable Battery for the Assessment of Neuropsychological Status; BCST—Berg Card Sorting Test.

### FAAH Activity Was Not Different Between Groups

**Figure 1.**
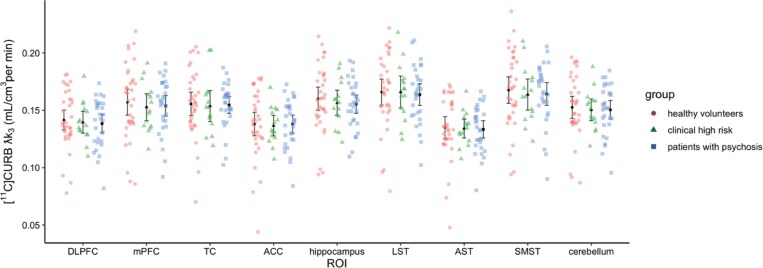
[^11^C]CURB λk_3_ in Healthy Controls, Clinical High Risk for Psychosis and Patients With Psychosis; DLPFC, Dorsolateral Prefrontal Cortex; mPFC, Medial Prefrontal Cortex; TC, Temporal Cortex; ACC, Anterior Cingulate Cortex; LST, Limbic Striatum; AST, Associative Striatum; SMST, Sensorimotor Striatum.

In agreement with our previous work^26^ there was no significant difference in brain [^11^C]CURB λk_3_ (FAAH activity) when comparing all 3 groups (HC, FEP, and CHR) (*F*_2, 75_ = 0.75, *P* = .48 (Cohen’s *f *= 0.141; small effect); ROI effect: *F*_8, 616_ = 146.74, *P* < .0001; *FAAH* rs324420 genotype effect: *F*_2, 75_ = 37.32, *P* < .0001; group × ROI interaction effect: *F*_16, 616_ = 0.41, *P* = .98, Figure 1). The results remained unchanged when controlling for possible confounders (see Supplementary Materials).

### FAAH Activity Was Associated With Visuospatial Construction Between Groups

**Figure 2.**
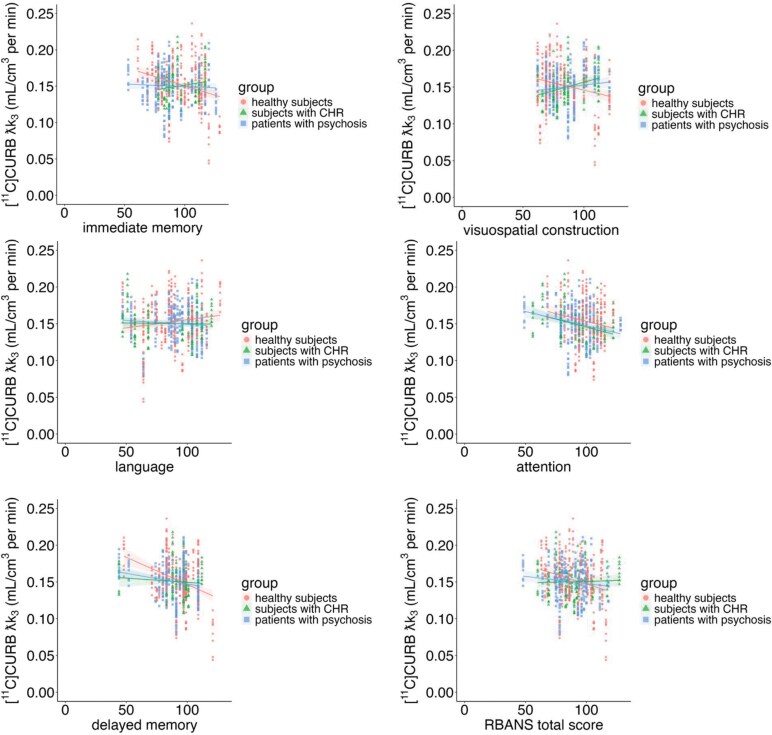
[^11^C]CURB λk_3_ and Cognitive Performance in Healthy Controls, Clinical High Risk for Psychosis and Patients With Psychosis with All ROIs Combined (ie, as There Was No Group × Cognitive Performance × ROI Effect). Post Hoc Partial Correlations Including Genotype Revealed That There Was a Negative Association Between FAAH Activity and Visuospatial Construction in HC (*r* = −0.25, *P* = .00006), While the Relationship Was Positive in CHR Individuals (*r* = 0.34, *P* = .00006) and in FEP (*r* = 0.23, *P* = .0001).

Overall there was a difference between groups in the association between RBANS visuospatial construction and FAAH activity. Furthermore, FAAH activity was also associated with BCST perseverative responses % but not with RBANS total score, immediate memory, attention, language, and delayed memory (see Figure 2 and Supplementary Materials).

Visuospatial construction—The association between FAAH activity and visuospatial construction was different by group (group × RBANS visuospatial construction *F*_2,72_ = 4.61, *P* = .01; Cohen’s *f* = .36; medium effect), such that lower FAAH was associated with better cognitive scores in HC but not in CHR or FEP groups (Figure 1); group (*F*_2, 72_ = 5.19, *P* = .007), ROI (*F*_8, 592_ = 3.98, *P* < .0001), and *FAAH* rs324420 genotype (*F*_2, 72_ = 40.73, *P* < .0001). The results remained unchanged when covariates were included (see Supplementary Materials).

### FAAH Activity Was Associated With Cognitive Flexibility Across Groups

**Figure 3.**
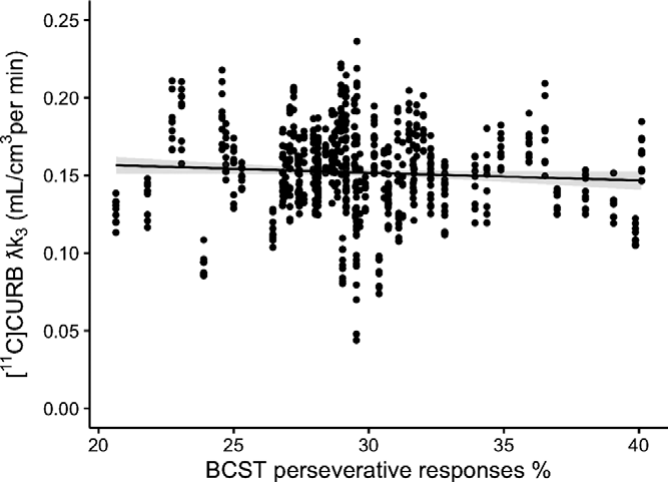
[^11^C]CURB λk3 and BCST Perseverative Response % in Healthy Controls, Clinical High Risk for Psychosis, and Patients With Psychosis With All ROIs Combined (ie, as there was no group × Score × ROI Effect).

There was a significant association between FAAH activity and percentage perseverative responses (*F*_1, 66_ = 5.09, *P* = .03; Cohen’s *f* = 0.28, medium effect) but not BCST error percentage (see Figure 3 and Supplementary Materials), such that lower FAAH was associated with greater percentage perseverative responses, regardless of group (*F*_2, 66_ = 2.12, *P* = .13), with significant ROI (*F*_8, 584_ = 155.34, *P* < .0001) and *FAAH* rs324420 genotype (*F*_2, 66_ = 37.19, *P* < .0001). The results remained unchanged when covariates were included (see Supplementary Materials).

## Discussion

This is the first work to demonstrate that brain FAAH activity is associated with specific domains of cognition (visuospatial construction and cognitive flexibility) but not overall cognitive performance (RBANS total). While the correlation between RBANS visuospatial construction and FAAH activity was negative in the HC group (lower FAAH activity is associated with better cognition), it was positive in the FEP and CHR groups. Furthermore, lower FAAH was associated with a greater percentage of perseverative responses (poorer cognitive flexibility) across all groups. These results were not impacted by possible confounders such as age, sex, years of education, medication status, smoking status, or cannabis exposure.

We did not detect a difference in FAAH activity between the 3 groups, consistent with findings from Watts et al.^26^ Regarding the relationship between FAAH activity and positive symptoms, the same positive association was only found when correcting for antipsychotic dose in the FEP cohort. In the CHR group, we did not find any relationship to prodromal symptoms. The complex regulation of different neurotransmitter systems by endocannabinoids might be responsible for the distinctive impact of endocannabinoids on different symptom domains (psychosis and cognitive) in psychosis.^15^ First, it has been proposed that increased anandamide measured in the acute phase of psychosis might be a regulatory response of the brain to activate neuroplasticity-related pathways.^61^ Consequently, FAAH activity and the potential consequence in anandamide levels might reflect an adaptive response that supports cognition in FEP and CHR groups. In contrast, the same regulatory mechanism may contribute to poorer cognitive performance in HCs, where no such compensatory response is required.

To some extent, this 2-way action is mirrored by cannabis effects in patients with psychosis, in which further cognitive impairment by regular cannabis use may even be beneficial on cognition,^62–64^ while at the same time, positive symptoms were more present in cannabis-using patients compared to patients abstinent from cannabis.^65^

Our data suggest that better cognitive flexibility, as seen in lower percent perseverative responses, is associated with greater FAAH activity across all groups. It might be indicative of lower anandamide levels that exert regulatory effects in striatal and cortical regions key for cognition, as was shown in rodents.^66^ But can we thus conclude that anandamide alone might have a cognition-impairing effect? The evidence from studies has been inconclusive in this regard. Nevertheless, one rodent study specifically showed that THC diminished prefrontal anandamide levels with reduced cognitive performance.^67^ Furthermore, anandamide’s involvement in cognition could be through the regulation of other neurotransmitter systems.^15^ For instance, the endocannabinoid system interacts with dopamine and glutamate, which are involved in cognitive processes.^66,68,69^ The especially robust relationship of FAAH activity and perseverative response scores might be speculated to be due to the fact that CB1 receptors are mainly located on glutamatergic pyramidal neurons and interneurons of the human cortex,^70,71^ while the association with visuospatial constructional (figure copy and line orientation) might be reflective of the endocannabinoids’ involvement in short-term and long-term synaptic cortical plasticity.^72^ In rodents, it was observed that anandamide facilitates learning processes in the hippocampus by modulating glutamate^66^ and GABA signaling via presynaptic sites.^73^ In accordance, Watts and co-authors reported an association between higher brain FAAH activity and higher levels of hippocampal Glx and smaller hippocampal volume.^74^ In striatal regions, for which we also observed robust relationships regarding FAAH activity and cognitive flexibility, endocannabinoids modulate dopamine-driven excitatory currents in rodents.^68,75^ Thus, it can be speculated that the role of FAAH and endocannabinoids on cognition occurs via glutamate or dopamine-based signaling next to other possible yet unknown mechanisms. Next, we aim to hypothesize that cognitive tasks assessing different domains may vary in their sensitivity to FAAH levels. It is possible that tasks involving memory and attention are less responsive to changes in FAAH expression or are influenced by distinct mechanisms compared to visuospatial construction and cognitive flexibility tasks. In addition, unique patterns of FAAH expression or endocannabinoid signaling in specific brain regions may contribute, as ROI significantly predicted FAAH.

The following limitations of the study must be addressed. First, we did not measure anandamide levels. However, the assumption of higher [^11^C]CURB λk_3_ leading to lower anandamide is supported by an existing human study measuring both parameters in abstinent patients with alcohol use disorder.^76^ This was confirmed in a rodent model.^77–79^ Another limitation is the low number of female participants especially in FEP partially a consequence of a slightly lower incidence of psychotic disorders in females.^1^ As there are known differences in the endocannabinoid system of male and female individuals^33,80^ and cognitive deficits also appear to be subject to sex effects,^81^ future studies should put a focus on this aspect. Next, lifetime cannabis exposure was higher in patients (see Table 1), even if the difference was only present on a trend level. Cannabis consumption is known to downregulate FAAH activity in young individuals^82^ and CB1 receptors in chronic cannabis smokers.^83^ Even though we excluded participants with a positive cannabis urine drug screen and individuals with a manifest cannabis use disorder, the total amount of lifetime cannabis use in FEP was higher. While past year cannabis use or lifetime cannabis use was considered as a covariate in the statistical tests it still cannot be completely ruled out that our results are partly explained by this phenomenon, since the responses rely on self-report. Furthermore, the present CHR group represents a unique sample of individuals with CHR who did not meet the criteria for concurrent DSM-IV axis 1 comorbidities (such as MDD, anxiety, etc.) which are highly prevalent in this population. This warrants follow-up studies of CHR, given the heterogeneity within CHR cohorts in transitioning to psychosis.^84,85^ Finally, future studies should address the potential role of FAAH inhibitors or cannabidiol on cognition in patients with schizophrenia, as the latter might have beneficial effects for FEP through FAAH modulation.^86,87^ Next, the mechanisms of 2-arachidonoyl glycerol (2-AG) and other endocannabinoid-metabolizing enzymes such as diacylglycerol lipase (DAGL) or monoacylglycerol lipase (MAGL) have to be more closely inspected as well.^15^

## Conclusion

While there was no difference in FAAH activity between groups, our results support a relationship between domain-specific cognitive performance and FAAH; with a group-dependent relationship between FAAH and visuospatial construction domain and a negative relationship between FAAH with perseverative responses across all groups. FAAH activity possibly regulates visuospatial cognition by modulating other neurotransmitters throughout the brain and this regulatory mechanism might be compromised in CHR and FEP. This should motivate future research regarding FAAH modulation for the improvement of cognitive symptoms in patients with neuropsychiatric disorders.

## Supplementary Material

Supplementary material is available at https://academic.oup.com/schizophreniabulletin/.

sbae212_suppl_Supplementary_Material

## Notes

A. Weidenauer and R. Garani contributed equally to this work.

## References

[1] Jauhar S, Johnstone M, McKenna PJ. Schizophrenia. Lancet. 2022;399:473–486. https://doi.org/10.1016/S0140-6736(21)01730-X 35093231

[2] Siskind D, Orr S, Sinha S, et al Rates of treatment-resistant schizophrenia from first-episode cohorts: systematic review and meta-analysis. Br J Psychiatry. 2022;220:115–120. https://doi.org/10.1192/bjp.2021.61 35049446

[3] Reichenberg A, Caspi A, Harrington H, et al Static and dynamic cognitive deficits in childhood preceding adult schizophrenia: a 30-year study. Am J Psychiatry. 2010;167:160–169. https://doi.org/10.1176/appi.ajp.2009.09040574 20048021 PMC3552325

[4] Carbon M, Correll CU. Thinking and acting beyond the positive: the role of the cognitive and negative symptoms in schizophrenia. CNS Spectr. 2014;19:35–53. https://doi.org/10.1017/S1092852914000601 25403863

[5] Conn KA, Burne THJ, Kesby JP. Subcortical dopamine and cognition in schizophrenia: looking beyond psychosis in preclinical models. Front Neurosci. 2020;14:542. https://doi.org/10.3389/fnins.2020.00542 32655348 PMC7325949

[6] Kegeles LS, Abi-Dargham A, Frankle WG, et al Increased synaptic dopamine function in associative regions of the striatum in schizophrenia. Arch Gen Psychiatry. 2010;67:231–239. https://doi.org/10.1001/archgenpsychiatry.2010.10 20194823

[7] Rao N, Northoff G, Tagore A, et al Impaired prefrontal cortical dopamine release in schizophrenia during a cognitive task: a [11C]FLB 457 positron emission tomography study. Schizophr Bull. 2019;45:670–679. https://doi.org/10.1093/schbul/sby076 29878197 PMC6483585

[8] Sakurai H, Bies RR, Stroup ST, et al Dopamine D2 receptor occupancy and cognition in schizophrenia: analysis of the CATIE data. Schizophr Bull. 2013;39:564–574. https://doi.org/10.1093/schbul/sbr189 22290266 PMC3627781

[9] Kano M, Ohno-Shosaku T, Hashimotodani Y, Uchigashima M, Watanabe M. Endocannabinoid-mediated control of synaptic transmission. Physiol Rev. 2009;89:309–380. https://doi.org/10.1152/physrev.00019.2008 19126760

[10] Katona I, Freund TF. Endocannabinoid signaling as a synaptic circuit breaker in neurological disease. Nat Med. 2008;14:923–930. https://doi.org/10.1038/nm.f.1869 18776886

[11] Pertwee RG. Cannabinoid pharmacology: the first 66 years. Br J Pharmacol. 2006;147:S163–S171. https://doi.org/10.1038/sj.bjp.0706406 16402100 PMC1760722

[12] Pertwee RG, Howlett AC, Abood ME, et al International union of basic and clinical pharmacology. LXXIX. Cannabinoid receptors and their ligands: beyond CB₁ and CB₂. Pharmacol Rev. 2010;62:588–631. https://doi.org/10.1124/pr.110.003004 21079038 PMC2993256

[13] Pertwee RG, Ross RA. Cannabinoid receptors and their ligands. Prostaglandins Leukot Essent Fatty Acids. 2002;66:101–121. https://doi.org/10.1054/plef.2001.0341 12052030

[14] Garani R, Watts JJ, Mizrahi R. Endocannabinoid system in psychotic and mood disorders, a review of human studies. Prog Neuropsychopharmacol Biol Psychiatry. 2021;106:110096. https://doi.org/10.1016/j.pnpbp.2020.110096 32898588 PMC8582009

[15] Lu HC, Mackie K. An introduction to the endogenous cannabinoid system. Biol Psychiatry. 2016;79:516–525. https://doi.org/10.1016/j.biopsych.2015.07.028 26698193 PMC4789136

[16] Hillard CJ. The endocannabinoid signaling system in the CNS: a primer. Int Rev Neurobiol. 2015;125:1–47. https://doi.org/10.1016/bs.irn.2015.10.001 26638763 PMC6813823

[17] Ceccarini J, De Hert M, Van Winkel R, et al Increased ventral striatal CB1 receptor binding is related to negative symptoms in drug-free patients with schizophrenia. Neuroimage. 2013;79:304–312. https://doi.org/10.1016/j.neuroimage.2013.04.052 23624489

[18] Wong DF, Kuwabara H, Horti A, et al Quantification of cerebral cannabinoid receptors subtype 1 (CB1) in healthy subjects and schizophrenia by the novel PET radioligand [11C]OMAR. Neuroimage. 2010;52:1505–1513. https://doi.org/10.1016/j.neuroimage.2010.04.034 20406692 PMC6580862

[19] Borgan F, Beck K, Butler E, et al The effects of cannabinoid 1 receptor compounds on memory: a meta-analysis and systematic review across species. Psychopharmacology (Berl). 2019;236:3257–3270. https://doi.org/10.1007/s00213-019-05283-3 31165913 PMC6828623

[20] Ceccarini J, Hompes T, Verhaeghen A, et al Changes in cerebral CB1 receptor availability after acute and chronic alcohol abuse and monitored abstinence. J Neurosci. 2014;34:2822–2831. https://doi.org/10.1523/JNEUROSCI.0849-13.2014 24553924 PMC6608522

[21] Ranganathan M, Cortes-Briones J, Radhakrishnan R, et al Reduced brain cannabinoid receptor availability in schizophrenia. Biol Psychiatry. 2016;79:997–1005. https://doi.org/10.1016/j.biopsych.2015.08.021 26432420 PMC4884543

[22] Appiah-Kusi E, Wilson R, Colizzi M, et al Childhood trauma and being at-risk for psychosis are associated with higher peripheral endocannabinoids. Psychol Med. 2020;50:1862–1871. https://doi.org/10.1017/S0033291719001946 31422779

[23] Giuffrida A, Leweke FM, Gerth CW, et al Cerebrospinal anandamide levels are elevated in acute schizophrenia and are inversely correlated with psychotic symptoms. Neuropsychopharmacology. 2004;29:2108–2114. https://doi.org/10.1038/sj.npp.1300558 15354183

[24] Koethe D, Giuffrida A, Schreiber D, et al Anandamide elevation in cerebrospinal fluid in initial prodromal states of psychosis. Br J Psychiatry. 2009;194:371–372. https://doi.org/10.1192/bjp.bp.108.053843 19336792

[25] Wang D, Sun X, Yan J, et al Alterations of eicosanoids and related mediators in patients with schizophrenia. J Psychiatr Res. 2018;102:168–178. https://doi.org/10.1016/j.jpsychires.2018.04.002 29674269

[26] Watts JJ, Jacobson MR, Lalang N, et al Imaging brain fatty acid amide hydrolase in untreated patients with psychosis. Biol Psychiatry. 2020;88:727–735. https://doi.org/10.1016/j.biopsych.2020.03.003 32387132 PMC8240477

[27] Boggs DL, Surti T, Gupta A, et al The effects of cannabidiol (CBD) on cognition and symptoms in outpatients with chronic schizophrenia a randomized placebo controlled trial. Psychopharmacology (Berl). 2018;235:1923–1932. https://doi.org/10.1007/s00213-018-4885-9 29619533

[28] McGuire P, Robson P, Cubala WJ, et al Cannabidiol (CBD) as an adjunctive therapy in schizophrenia: a multicenter randomized controlled trial. Am J Psychiatry. 2018;175:225–231. https://doi.org/10.1176/appi.ajp.2017.17030325 29241357

[29] Hotz J, Fehlmann B, Papassotiropoulos A, de Quervain DJ, Schicktanz NS. Cannabidiol enhances verbal episodic memory in healthy young participants: a randomized clinical trial. J Psychiatr Res. 2021;143:327–333. https://doi.org/10.1016/j.jpsychires.2021.09.007 34536664

[30] Bioque M, Cabrera B, García-Bueno B, et al; FLAMM-PEPs Study - Centro de Investigación Biomédica en Red de Salud Mental. Dysregulated peripheral endocannabinoid system signaling is associated with cognitive deficits in first-episode psychosis. J Psychiatr Res. 2016;75:14–21. https://doi.org/10.1016/j.jpsychires.2016.01.002 26783729

[31] Ferretjans R, de Campos SM, Ribeiro-Santos R, et al Cognitive performance and peripheral endocannabinoid system receptor expression in schizophrenia. Schizophr Res. 2014;156:254–260. https://doi.org/10.1016/j.schres.2014.04.028 24853061

[32] Borgan F, Laurikainen H, Veronese M, et al; METSY Group. In vivo availability of cannabinoid 1 receptor levels in patients with first-episode psychosis. JAMA Psychiatry. 2019;76:1074–1084. https://doi.org/10.1001/jamapsychiatry.2019.1427 31268519 PMC6613300

[33] Laurikainen H, Tuominen L, Tikka M, et al; METSY group. Sex difference in brain CB1 receptor availability in man. Neuroimage. 2019;184:834–842. https://doi.org/10.1016/j.neuroimage.2018.10.013 30296558

[34] Jung KM, Astarita G, Yasar S, et al An amyloid β42-dependent deficit in anandamide mobilization is associated with cognitive dysfunction in Alzheimer’s disease. Neurobiol Aging. 2012;33:1522–1532. https://doi.org/10.1016/j.neurobiolaging.2011.03.012 21546126 PMC3154439

[35] Li GL, Winter H, Arends R, et al Assessment of the pharmacology and tolerability of PF-04457845, an irreversible inhibitor of fatty acid amide hydrolase-1, in healthy subjects. Br J Clin Pharmacol. 2012;73:706–716. https://doi.org/10.1111/j.1365-2125.2011.04137.x 22044402 PMC3403198

[36] Bedse G, Bluett RJ, Patrick TA, et al Therapeutic endocannabinoid augmentation for mood and anxiety disorders: comparative profiling of FAAH, MAGL and dual inhibitors. Transl Psychiatry. 2018;8:92. https://doi.org/10.1038/s41398-018-0141-7 29695817 PMC5917016

[37] Kangas BD, Leonard MZ, Shukla VG, et al Comparisons of 9-Tetrahydrocannabinol and anandamide on a battery of cognition-related behavior in Nonhuman Primates. J Pharmacol Exp Ther. 2016;357:125–133. https://doi.org/10.1124/jpet.115.228189 26826191 PMC4809315

[38] Panlilio LV, Thorndike EB, Nikas SP, et al Effects of fatty acid amide hydrolase (FAAH) inhibitors on working memory in rats. Psychopharmacology (Berl). 2016;233:1879–1888. https://doi.org/10.1007/s00213-015-4140-6 26558620 PMC4846548

[39] Hasanein P, Teimuri Far M. Effects of URB597 as an inhibitor of fatty acid amide hydrolase on WIN55, 212-2-induced learning and memory deficits in rats. Pharmacol Biochem Behav. 2015;131:130–135. https://doi.org/10.1016/j.pbb.2015.02.007 25689415

[40] Hlavacova N, Chmelova M, Danevova V, Csanova A, Jezova D. Inhibition of fatty-acid amide hydrolyse (FAAH) exerts cognitive improvements in male but not female rats. Endocr Regul. 2015;49:131–136. https://doi.org/10.4149/endo_2015_03_131 26238495

[41] Jankovic M, Spasojevic N, Ferizovic H, Stefanovic B, Dronjak S. Sex specific effects of the fatty acid amide hydrolase inhibitor URB597 on memory and brain β2-adrenergic and D1-dopamine receptors. Neurosci Lett. 2022;768:136363. https://doi.org/10.1016/j.neulet.2021.136363 34843876

[42] Mazzola C, Medalie J, Scherma M, et al Fatty acid amide hydrolase (FAAH) inhibition enhances memory acquisition through activation of PPAR-alpha nuclear receptors. Learn Mem. 2009;16:332–337. https://doi.org/10.1101/lm.1145209 19403796 PMC2683005

[43] Rivera P, Fernández-Arjona MDM, Silva-Peña D, et al Pharmacological blockade of fatty acid amide hydrolase (FAAH) by URB597 improves memory and changes the phenotype of hippocampal microglia despite ethanol exposure. Biochem Pharmacol. 2018;157:244–257. https://doi.org/10.1016/j.bcp.2018.08.005 30098312

[44] Wang DP, Lin Q, Kang K, Wu YF, Su SH, Hai J. Preservation of spatial memory and neuroprotection by the fatty acid amide hydrolase inhibitor URB597 in a rat model of vascular dementia. Ann Transl Med. 2021;9:228. https://doi.org/10.21037/atm-20-4431 33708855 PMC7940933

[45] Miller TJ, McGlashan TH, Rosen JL, et al Prodromal assessment with the structured interview for prodromal syndromes and the scale of prodromal symptoms: predictive validity, interrater reliability, and training to reliability. Schizophr Bull. 2003;29:703–715. https://doi.org/10.1093/oxfordjournals.schbul.a007040 14989408

[46] Andreasen NC, Pressler M, Nopoulos P, Miller D, Ho BC. Antipsychotic dose equivalents and dose-years: a standardized method for comparing exposure to different drugs. Biol Psychiatry. 2010;67:255–262. https://doi.org/10.1016/j.biopsych.2009.08.040 19897178 PMC3677042

[47] Randolph C, Tierney MC, Mohr E, Chase TN. The repeatable battery for the assessment of neuropsychological status (RBANS): preliminary clinical validity. J Clin Exp Neuropsychol. 1998;20:310–319. https://doi.org/10.1076/jcen.20.3.310.823 9845158

[48] Gold JM, Queern C, Iannone VN, Buchanan RW. Repeatable battery for the assessment of neuropsychological status as a screening test in schizophrenia I: sensitivity, reliability, and validity. Am J Psychiatry. 1999;156:1944–1950. https://doi.org/10.1176/ajp.156.12.1944 10588409

[49] Fox CJ, Mueller ST, Gray HM, Raber J, Piper BJ. Evaluation of a short-form of the berg card sorting test. PLoS One. 2013;8:e63885. https://doi.org/10.1371/journal.pone.0063885 23691107 PMC3653789

[50] Mueller ST, Piper BJ. The psychology experiment building language (PEBL) and PEBL test battery. J Neurosci Methods. 2014;222:250–259. https://doi.org/10.1016/j.jneumeth.2013.10.024 24269254 PMC3897935

[51] Chiu EC, Lee SC. Test–retest reliability of the Wisconsin card sorting test in people with schizophrenia. Disabil Rehabil. 2019;43:996–1000. https://doi.org/10.1080/09638288.2019.1647295 31361972

[52] Tyburski E, Mak M, Samochowiec A, et al The relationship between cingulum bundle integrity and different aspects of executive functions in chronic schizophrenia. Prog Neuropsychopharmacol Biol Psychiatry. 2020;102:109955. https://doi.org/10.1016/j.pnpbp.2020.109955 32360815

[53] Kay SR, Fiszbein A, Opler LA. The positive and negative syndrome scale (PANSS) for schizophrenia. Schizophr Bull. 1987;13:261–276. https://doi.org/10.1093/schbul/13.2.261 3616518

[54] Lehoux C, Gobeil MH, Lefèbvre AA, Maziade M, Roy MA. The five-factor structure of the PANSS: a critical review of its consistency across studies. Clin Schizophr Related Psychoses. 2009;3:103–110. https://doi.org/10.3371/CSRP.3.2.5

[55] Miller TJ, McGlashan TH, Woods SW, et al Structured interview for prodromal symptoms. 1999. https://doi.org/10.1037/t15939-000

[56] Cole MW, Schneider W. The cognitive control network: integrated cortical regions with dissociable functions. Neuroimage. 2007;37:343–360. https://doi.org/10.1016/j.neuroimage.2007.03.071 17553704

[57] Rusjan P, Mamo D, Ginovart N, et al An automated method for the extraction of regional data from PET images. Psychiatry Res. 2006;147:79–89. https://doi.org/10.1016/j.pscychresns.2006.01.011 16797168

[58] Rusjan PM, Wilson AA, Mizrahi R, et al Mapping human brain fatty acid amide hydrolase activity with PET. J Cereb Blood Flow Metab 2013;33:407–414. https://doi.org/10.1038/jcbfm.2012.180 23211960 PMC3587811

[59] Boileau I, Tyndale RF, Williams B, et al The fatty acid amide hydrolase C385A variant affects brain binding of the positron emission tomography tracer [^11^ C]CURB. J Cereb Blood Flow Metab. 2015;35:1237–1240. https://doi.org/10.1038/jcbfm.2015.119 26036940 PMC4527995

[60] R Working Group. A Language and Environment for Statistical Computing. R Foundation for Statistical Computing, 2021.

[61] Castillo PE, Younts TJ, Chávez AE, Hashimotodani Y. Endocannabinoid signaling and synaptic function. Neuron. 2012;76:70–81. https://doi.org/10.1016/j.neuron.2012.09.020 23040807 PMC3517813

[62] Cunha PJ, Rosa PGP, Ayres A de M, et al Cannabis use, cognition and brain structure in first-episode psychosis. Schizophr Res. 2013;147:209–215. https://doi.org/10.1016/j.schres.2013.04.009 23672820

[63] de Vos C, Leopold K, Blanke ES, et al The relationship between cannabis use and cognition in people diagnosed with first-episode psychosis. Psychiatry Res. 2020;293:113424. https://doi.org/10.1016/j.psychres.2020.113424 32862065

[64] Power BD, Dragovic M, Badcock JC, et al No additive effect of cannabis on cognition in schizophrenia. Schizophr Res. 2015;168:245–251. https://doi.org/10.1016/j.schres.2015.06.026 26235754

[65] Pope LG, Manseau MW, Kelley ME, Compton MT. Symptomatology and neurocognition among first-episode psychosis patients with and without cannabis use in the three months prior to first hospitalization. Schizophr Res. 2021;228:83–88. https://doi.org/10.1016/j.schres.2020.12.012 33434738

[66] Zimmermann T, Bartsch JC, Beer A, et al Impaired anandamide/palmitoylethanolamide signaling in hippocampal glutamatergic neurons alters synaptic plasticity, learning, and emotional responses. Neuropsychopharmacology. 2019;44:1377–1388. https://doi.org/10.1038/s41386-018-0274-7 30532004 PMC6784910

[67] Vigano D, Guidali C, Petrosino S, et al Involvement of the endocannabinoid system in phencyclidine-induced cognitive deficits modelling schizophrenia. Int J Neuropsychopharmacol. 2009;12:599–614. https://doi.org/10.1017/S1461145708009371 18789179

[68] André VM, Cepeda C, Cummings DM, et al Dopamine modulation of excitatory currents in the striatum is dictated by the expression of D1 or D2 receptors and modified by endocannabinoids. Eur J Neurosci. 2010;31:14–28. https://doi.org/10.1111/j.1460-9568.2009.07047.x 20092552

[69] Ceccarini J, Koole M, Van Laere K. Cannabinoid receptor availability modulates the magnitude of dopamine release in vivo in the human reward system: a preliminary multitracer positron emission tomography study. Addict Biol. 2022;27:e13167. https://doi.org/10.1111/adb.13167 35470551

[70] Hill EL, Gallopin T, Férézou I, et al Functional CB1 receptors are broadly expressed in neocortical GABAergic and glutamatergic neurons. J Neurophysiol. 2007;97:2580–2589. https://doi.org/10.1152/jn.00603.2006 17267760

[71] Marsicano G, Lutz B. Expression of the cannabinoid receptor CB1 in distinct neuronal subpopulations in the adult mouse forebrain. Eur J Neurosci. 1999;11:4213–4225. https://doi.org/10.1046/j.1460-9568.1999.00847.x 10594647

[72] Durieux LJA, Gilissen SRJ, Arckens L. Endocannabinoids and cortical plasticity: CB1R as a possible regulator of the excitation/inhibition balance in health and disease. Eur J Neurosci. 2022;55:971–988. https://doi.org/10.1111/ejn.15110 33427341

[73] Lemtiri-Chlieh F, Levine ES. 2-AG and anandamide enhance hippocampal long-term potentiation via suppression of inhibition. Front Cell Neurosci. 2022;16:1023541. https://doi.org/10.3389/fncel.2022.1023541 36212685 PMC9534525

[74] Watts JJ, Guma E, Chavez S, et al In vivo brain endocannabinoid metabolism is related to hippocampus glutamate and structure—a multimodal imaging study with PET, 1H-MRS, and MRI. Neuropsychopharmacology. 2022;47:1984–1991. https://doi.org/10.1038/s41386-022-01384-4 35906490 PMC9485131

[75] Solinas M, Justinova Z, Goldberg SR, Tanda G. Anandamide administration alone and after inhibition of fatty acid amide hydrolase (FAAH) increases dopamine levels in the nucleus accumbens shell in rats. J Neurochem. 2006;98:408–419. https://doi.org/10.1111/j.1471-4159.2006.03880.x 16805835

[76] Best LM, Williams B, Le Foll B, et al Lower brain fatty acid amide hydrolase in treatment-seeking patients with alcohol use disorder: a positron emission tomography study with [C-11]CURB. Neuropsychopharmacology. 2020;45:1289–1296. https://doi.org/10.1038/s41386-020-0606-2 31910433 PMC7298050

[77] Cippitelli A, Cannella N, Braconi S, et al Increase of brain endocannabinoid anandamide levels by FAAH inhibition and alcohol abuse behaviours in the rat. Psychopharmacology (Berl). 2008;198:449–460. https://doi.org/10.1007/s00213-008-1104-0 18446329

[78] Cravatt BF, Demarest K, Patricelli MP, et al Supersensitivity to anandamide and enhanced endogenous cannabinoid signaling in mice lacking fatty acid amide hydrolase. Proc Natl Acad Sci U S A. 2001;98:9371–9376. https://doi.org/10.1073/pnas.161191698 11470906 PMC55427

[79] Dincheva I, Drysdale AT, Hartley CA, et al FAAH genetic variation enhances fronto-amygdala function in mouse and human. Nat Commun. 2015;6:6395. https://doi.org/10.1038/ncomms7395 25731744 PMC4351757

[80] Craft RM, Marusich JA, Wiley JL. Sex differences in cannabinoid pharmacology: a reflection of differences in the endocannabinoid system? Life Sci. 2013;92:476–481. https://doi.org/10.1016/j.lfs.2012.06.009 22728714 PMC3492530

[81] Mendrek A, Mancini-Marïe A. Sex/gender differences in the brain and cognition in schizophrenia. Neurosci Biobehav Rev. 2016;67:57–78. https://doi.org/10.1016/j.neubiorev.2015.10.013 26743859

[82] Jacobson MR, Watts JJ, Da Silva T, et al Fatty acid amide hydrolase is lower in young cannabis users. Addict Biol. 2021;26:e12872. https://doi.org/10.1111/adb.12872 31960544 PMC7944390

[83] Hirvonen J, Goodwin R, Li CT, et al Reversible and regionally selective downregulation of brain cannabinoid CB1 receptors in chronic daily cannabis smokers. Mol Psychiatry. 2012;17:642–649. https://doi.org/10.1038/mp.2011.82 21747398 PMC3223558

[84] Salazar de Pablo G, Radua J, Pereira J, et al Probability of transition to psychosis in individuals at clinical high risk: an updated meta-analysis. JAMA Psychiatry. 2021;78:970–978. https://doi.org/10.1001/jamapsychiatry.2021.0830 34259821 PMC8281006

[85] Salazar de Pablo G, Soardo L, Cabras A, et al Clinical outcomes in individuals at clinical high risk of psychosis who do not transition to psychosis: a meta-analysis. Epidemiol Psychiatr Sci. 2022;31:e9. https://doi.org/10.1017/S2045796021000639 35042573 PMC8786617

[86] Khoury JM, Neves MdeCLdas, Roque MAV, et al Is there a role for cannabidiol in psychiatry? World J Biol Psychiatry. 2019;20:101–116. https://doi.org/10.1080/15622975.2017.1285049 28112021

[87] Leweke FM, Piomelli D, Pahlisch F, et al Cannabidiol enhances anandamide signaling and alleviates psychotic symptoms of schizophrenia. Transl Psychiatry. 2012;2:e94–e94. https://doi.org/10.1038/tp.2012.15 22832859 PMC3316151

