# Supplementary material for: The Role of Fatty Acid Amide Hydrolase, a Key Regulatory Endocannabinoid Enzyme, in Domain-Specific Cognitive Performance in Psychosis
Source: Schizophr Bull. 2024 Dec 27;51(5):1328–38. doi: 10.1093/schbul/sbae212 (PMC12414559; doi:10.1093/schbul/sbae212)
Supplement: sbae212_suppl_Supplementary_Material [file sbae212_suppl_supplementary_material.docx]

**The Role of Fatty Acid Amide Hydrolase, a Key Regulatory Endocannabinoid Enzyme, in Domain Specific Cognitive Performance in Psychosis**

***Supplementary Information***

[Supplementary Methods 2](#_Toc154060688)

[Participants 2](#_Toc154060689)

[Supplementary results 4](#_Toc154060690)

[FAAH activity and cognitive performance between groups 4](#_Toc154060691)

[FAAH activity and cognitive flexibility across groups: Analysis after inclusion of extreme BCST outlier 7](#_Toc154060692)

[FAAH activity and cognitive performance between groups by regions of interest 8](#_Toc154060693)

[Cannabis exposure and cognition 10](#_Toc154060694)

# Supplementary methods

### Participants

The CaTS BioBank repository with retrospective [^11^C]CURB data includes current cannabis users (those that tested positive on urine THC drug screen) and non-users (those that tested negative on urine cannabis drug screen), individuals with psychotic disorder (n=57; that includes current cannabis users (n=12) and non-users (n=45)), clinical high risk for psychosis (that includes current cannabis users and non-users and healthy controls from collaborator (n=10).

Participants with current cannabis use (positive urine drug screens for cannabis), clinical high-risk groups with comorbidities are not described in the flow diagram below given inclusion/exclusion criteria for this specific study. Here, we have a total of n=177 participants who had a negative cannabis urine drug screen and who met inclusion criteria for the current study; of which, n=90 are healthy controls (including n=10 participants from collaborator), n=41 are individuals with clinical high risk for psychosis and n=45 are individuals with psychotic disorders.

(see CONSORT flow diagram).

All participants whose cognition data was available from Watts et al., 2020 (n=47; n=25 FEP, n=22 HC) have been included in this study. The Watts et al. 2020 study included a total of 63 participants, consisting of 27 individuals with a psychotic disorder and 36 healthy controls. In this study, we have expanded this cohort to include additional participants (n=33; HC=13, CHR=15, FEP=5, while excluding participants who did not have cognition data from the previous study (n=16; HC=14 and FEP =2).

**Excluded**

**[^11^C] CURB PET and cognition**

Met inclusion criteria at enrolment (n=177)

Healthy Control (n=80),
Healthy control from collaborator (n=10)

Psychotic disorder (n= 46)
FEP/SCZ

HC: Early discharges: discharged before MRI or [^11^C] CURB PET (n=39)

FEP: Early discharges: discharged before MRI or [^11^C] CURB PET (n=13)

Included HC (n= 35)

Included CHR (n=15)

**Completed study procedures and excluded**

high motion (n=1) or not genotyped (n=1)
no cognition data (n=14)

no cognition data (n=2),
diagnostic status did not meet eligibility at clinical follow-up (n=1)

Included FEP/SCZ (n=30)

Clinical High Risk (n=41)
CHR

CHR: Early discharges: discharged before MRI or [^11^C] CURB PET (n=25)

no cognition data (n=1)

**Figure S1**. CONSORT diagram showing participant selection.

# Supplementary results

### FAAH activity and cognitive performance between groups

*RBANS total score* – FAAH activity across all regions of interest was not associated with RBANS total score x group interaction (*F_2,72_*  = 1.46, *p* = 0.24), without a significant main effect of RBANS total score (*F_1, 72_*  = 0.16, *p* = 0.69) or main effect of group (*F_2, 72_*  = 1.79, *p* = 0.17), while controlling for ROI (*F_8,_* *_632_*  = 171.60, *p* <0.0001) and *FAAH* rs324420 genotype (*F_2, 72_* = 35.65, *p* <0.0001). The interaction effects were also not significant for group x RBANS total x ROI interactions (*F_16,592_*  = 1.00, *p* = 0.45).

*RBANS immediate memory* – FAAH activity across all regions of interest was not associated with RBANS immediate memory x group interaction (*F_2,72_*  = 1.74, *p* = 0.18), without a significant main effect of RBANS immediate memory (*F_1, 72_*  = 0.91, *p* = 0.34), or group (*F_2, 72_*  = 2.05, *p* = 0.14), controlling for ROI (*F_8,_* *_632_*  = 171.61, *p* <0.0001) and *FAAH* rs324420 genotype (*F_2, 72_* = 34.57, *p* <0.0001). There was also no significant group x RBANS immediate memory x ROI interaction (*F_16, 592_*  = 0.68, *p* = 0.81).

*RBANS language –* There was no association between FAAH activity across all regions of interest with RBANS language x group interaction (*F_2,_* *_72_*  = 0.50, *p* = 0.61), without a significant main effect of RBANS language (*F_1, 72_*  = 0.009, *p* = 0.93), or group (*F_2, 72_*  = 0.35, *p* = 0.70), while controlling for ROI (*F_8,_* *_632_*  = 171.60, *p* < 0.0001) and *FAAH* rs324420 genotype (*F_2, 72_* = 35.69, *p* <0.0001). There was also no group x RBANS language x ROI interaction (*F_16,_* *_592_*  = 0.96, *p* = 0.49).

*RBANS attention –* There was no significant association between FAAH activity across regions of interest with RBANS attention score x group interaction (*F_2, 72_*  = 0.59, *p* = 0.56), without a significant main effect of RBANS attention (*F_1, 72_*  = 2.29, *p* = 0.13), or group (*F_2, 72_*  = 0.95, *p* = 0.39), while controlling for ROI (*F_8,_* *_632_*  = 171.60, *p* <0.0001) and *FAAH* rs324420 genotype (*F_2, 72_*  = 36.04, *p* <0.0001). There was also no group x score x ROI interaction (*F_16, 592_*  = 1.07, *p* = 0.38).

*RBANS delayed memory –* There was no significant association between FAAH activity across regions of interest and RBANS delayed memory scores x group interaction (*F_2, 72_*  = 1.71, *p* = 0.18), without a significant main effect of RBANS delayed memory (*F_1, 72_*  = 1.06, *p* = 0.31), or group (*F_2, 72_*  = 1.96, *p* = 0.14), while controlling for ROI (*F_8,_* *_632_*  = 171.61, *p* <0.0001) and *FAAH* rs324420 genotype (*F_2, 72_*  = 34.24, *p* <0.0001). There was also no significant interaction effect of group x score x ROI interaction (*F_16, 592_*  = 0.49, *p* = 0.95).

*BCST errors % –*There was no significant association between FAAH activity and total percentage of errors in the BCST x group interaction (*F_2, 66_*  = 1.02, *p* = 0.37), without a significant main effect of BCST errors % (*F_1, 66_*  = 0.02, *p* = 0.88), or group (*F_2, 66_*  = 0.43, *p* = 0.65) while controlling for ROI (*F_8,_* *_584_*  = 155.34, *p* <0.0001) and *FAAH* rs324420 genotype (*F_2, 66_*  = 30.32, *p* <0.0001). There was also no significant group x score x ROI interaction (*F_16, 544_*  = 1.10, *p* = 0.35).

**FAAH activity and cognitive performance (RBANS) between groups and cognitive flexibility across groups with covariates**

| **Cognitive test**  **Covariate** | **Effect of group*score** | **effect of covariate** |
| --- | --- | --- |
| **Visuospatial construction** | ***F_2, 72_*  = 4.61, *p* = 0.01** |  |
| age | ***F_2, 71_*  = 4.62, *p* = 0.01** | *F_1, 71_*  = 0.61, *p* = 0.44 |
| sex | ***F_2, 71_*  = 4.89, *p* = 0.01** | ***F_1, 71_*  = 4.96, *p* = 0.03** |
| years of education | ***F_2, 71_* = 4.55, *p* = 0.01** | *F_1, 71_* = 0.08, *p* = 0.78 |
| antipsychotic dose | ***F_2, 71_* = 4.44, *p* = 0.01** | *F_1, 71_* = 0.04, *p* = 0.84 |
| cigarettes per day | ***F_2,71_*  = 4.49, *p* = 0.01** | *F_1, 71_* = 0.35, *p* = 0.55 |
| past year cannabis exposure | ***F_2, 71_* = 4.57, *p* = 0.01** | *F_1, 71_* = 0.33, *p* = 0.57 |
| cannabis lifetime exposure | ***F_2, 71_*  = 4.62, *p* = 0.01** | *F_1, 71_* = 0.18, *p* = 0.67 |
| **RBANS total** | *F_2, 72_*  = 1.46, *p* = 0.24 |  |
| age | *F_2, 71_*  = 1.44, *p* = 0.24 | *F_1, 71_*  = 0.66, *p* = 0.42 |
| sex | *F_2, 71_*  = 2.30, *p* = 0.10 | ***F_1, 71_*  = 6.02, *p* = 0.01** |
| years of education | *F_2, 71_*  = 1.45, *p* = 0.24 | *F_1, 71_*  = 0.03, *p* = 0.87 |
| antipsychotic dose | *F_2, 71_*  = 1.47, *p* = 0.24 | *F_1, 71_*  = 0.29, *p* = 0.59 |
| cigarettes per day | *F_2, 71_*  = 1.37, *p* = 0.26 | *F_1, 71_*  = 0.39, *p* = 0.53 |
| past year cannabis exposure | *F_2, 71_*  = 1.45, *p* = 0.24 | *F_1, 71_*  = 0.29, *p* = 0.59 |
| cannabis lifetime exposure | *F_2, 71_*  = 1.41, *p* = 0.25 | *F_1, 71_*  = 0.008, *p* = 0.93 |
| **RBANS immediate memory** | *F_2, 72_*  = 1.74, *p* = 0.18 |  |
| age | *F_2, 71_*  = 1.66, *p* = 0.19 | *F_1, 71_*  = 0.39, *p* = 0.53 |
| sex | *F_2, 71_*  = 2.09, *p* = 0.13 | *F_1, 71_*  = 4.95, *p* = 0.03 |
| years of education | *F_2, 71_*  = 1.85, *p* = 0.16 | *F_1, 71_*  = 0.32, *p* = 0.57 |
| antipsychotic dose | *F_2, 71_*  = 1.75, *p* = 0.18 | *F_1, 71_*  = 0.35, *p* = 0.56 |
| cigarettes per day | *F_2, 71_*  = 1.68, *p* = 0.19 | *F_1, 71_*  = 0.37, *p* = 0.54 |
| past year cannabis exposure | *F_2, 71_*  = 1.63, *p* = 0.20 | *F_1, 71_*  = 0.12, *p* = 0.74 |
| cannabis lifetime exposure | *F_2, 71_*  = 1.69, *p* = 0.19 | *F_1, 71_*  = 0.002, *p* = 0.96 |
| **RBANS language** | *F_2, 72_*  = 0.50, *p* = 0.60 |  |
| age | *F_2, 71_*  = 0.61, *p* = 0.55 | *F_1, 71_*  = 0.74, *p* = 0.39 |
| sex | *F_2, 71_*  = 0.29, *p* = 0.75 | *F_1, 71_*  = 3.75, *p* = 0.06 |
| years of education | *F_2, 71_*  = 0.48, *p* = 0.61 | *F_1, 71_*  = 0.04, *p* = 0.84 |
| antipsychotic dose | *F_2, 71_*  = 0.49, *p* = 0.61 | *F_1, 71_*  = 0.27, *p* = 0.60 |
| cigarettes per day | *F_2, 71_*  = 0.51, *p* = 0.60 | *F_1, 71_*  = 0.48, *p* = 0.49 |
| past year cannabis exposure | *F_2, 71_*  = 0.55, *p* = 0.58 | *F_1, 71_*  = 0.39, *p* = 0.53 |
| cannabis lifetime exposure | *F_2, 71_*  = 0.50, *p* = 0.60 | *F_1, 71_*  = 0.05, *p* = 0.82 |
| **RBANS attention** | *F_2, 72_*  = 0.59, *p* = 0.56 |  |
| age | *F_2, 71_*  = 0.58, *p* = 0.56 | *F_1, 71_*  = 0.54, *p* = 0.46 |
| sex | *F_2, 71_*  = 1.20, *p* = 0.31 | *F_1, 71_*  = 6.66, *p* = 0.01 |
| years of education | *F_2, 71_*  = 0.58, *p* = 0.56 | *F_1, 71_* = 0.001, *p* = 0.97 |
| antipsychotic dose | *F_2, 71_*  = 0.59, *p* = 0.56 | *F_1, 71_* = 0.23, *p* = 0.63 |
| cigarettes per day | *F_2, 71_*  = 0.57, *p* = 0.56 | *F_1, 71_* = 0.37, *p* = 0.54 |
| past year cannabis exposure | *F_2, 71_*  = 0.63, *p* = 0.53 | *F_1, 71_* = 0.24, *p* = 0.62 |
| cannabis lifetime exposure | *F_2, 71_*  = 0.57, *p* = 0.56 | *F_1, 71_* = 0.03, *p* = 0.86 |
| **RBANS delayed memory** | *F_2, 72_*  = 1.78, *p* = 0.18 |  |
| age | *F_2, 71_*  = 1.77, *p* = 0.18 | *F_1, 71_*  = 0.68, *p* = 0.41 |
| sex | *F_2, 71_*  = 2.73, *p* = 0.07 | *F_1, 71_*  = 6.12, *p* = 0.01 |
| years of education | *F_2, 71_*  = 1.69, *p* = 0.19 | *F_1, 71_* = 0.01, *p* = 0.93 |
| antipsychotic dose | *F_2, 71_*  = 1.79, *p* = 0.17 | *F_1, 71_* = 0.37, *p* = 0.54 |
| cigarettes per day | *F_2, 71_*  = 1.49, *p* = 0.23 | *F_1, 71_* = 0.35, *p* = 0.55 |
| past year cannabis exposure | *F_2, 71_*  = 1.75, *p* = 0.18 | *F_1, 71_* = 0.37, *p* = 0.54 |
| cannabis lifetime exposure | *F_2, 71_*  = 1.65, *p* = 0.19 | *F_1, 71_* = 0.01, *p* = 0.91 |
| **FAAH activity and cognitive flexibility across groups** | | |
| **BCST perseverative responses %** | **main effect of score** | **effect of covariate** |
| age | *F_1, 65_*  = 5.07, *p* = 0.03 | *F_1, 65_*  = 0.57, *p* = 0.45 |
| sex | *F_1, 65_*  = 4.87, *p* = 0.03 | *F_1, 65_*  = 2.24, *p* = 0.14 |
| years of education | *F_1, 65_* = 5.27, *p* = 0.02 | *F_1, 65_* = 0.33, *p* = 0.57 |
| antipsychotic dose | *F_1, 65_* = 4.89, *p* = 0.03 | *F_1, 65_* = 0.12, *p* = 0.73 |
| cigarettes per day | *F_1, 65_*  = 5.47, *p* = 0.02 | *F_1, 65_* = 0.73, *p* = 0.39 |
| past year cannabis exposure | *F_1, 65_* = 5.32, *p* = 0.02 | *F_1, 65_* = 0.49, *p* = 0.49 |
| cannabis lifetime exposure | *F_1, 65_*  = 5.05, *p* = 0.03 | *F_1, 65_* = 0.03, *p* = 0.85 |
| **BCST errors %** |  |  |
| age | *F_1, 65_*  = 0.03, *p* = 0.85 | *F_1, 65_*  = 1.02, *p* = 0.31 |
| sex | *F_1, 65_*  = 0.15, *p* = 0.70 | *F_1, 65_*  = 3.75, *p* = 0.05 |
| years of education | *F_1, 65_* = 0.03, *p* = 0.87 | *F_1, 65_* = 0.06, *p* = 0.80 |
| antipsychotic dose | *F_1, 65_* = 0.01, *p* = 0.91 | *F_1, 65_* = 0.21, *p* = 0.64 |
| cigarettes per day | *F_1, 65_*  = 0.06, *p* = 0.81 | *F_1, 65_* = 0.43, *p* = 0.51 |
| past year cannabis exposure | *F_1, 65_* = 0.02, *p* = 0.88 | *F_1, 65_* = 0.24, *p* = 0.62 |
| cannabis lifetime exposure | *F_1, 65_*  = 0.02, *p* = 0.88 | *F_1, 65_* = 0.0001, *p* = 0.99 |

**Table S1. Effect of each covariate on group*score and main effect of covariate in different domains of cognition.** ROI and genotype were controlled for and the interaction between study group and RBANS subscale scores (with separated models for different subscales).

### FAAH activity and cognitive flexibility across groups: Analysis after inclusion of extreme BCST outlier

*BCST perseverative responses % –* There was a significant association between FAAH activity and percentage of perseverative responses (*F_1, 67_*  = 6.80, *p* = 0.01), controlling for group (*F_2, 67_*  = 1.90, *p* = 0.16), ROI (*F_8,_* *_584_*  = 155.37, *p* <0.0001) and *FAAH* rs324420 genotype (*F_2, 67_*  = 37.30, *p* <0.0001), with no interaction effect of group by score (F_2,67_ = 2.29, p = 0.10) and no a significant group x score x ROI interaction (*F_16, 552_*  = 0.55, *p* = 0.91). The results remained unchanged when controlling for covariates (data not shown).

There were no significant associations between FAAH activity and BCST error % when the outlier was included (see **Figure S2**).

**
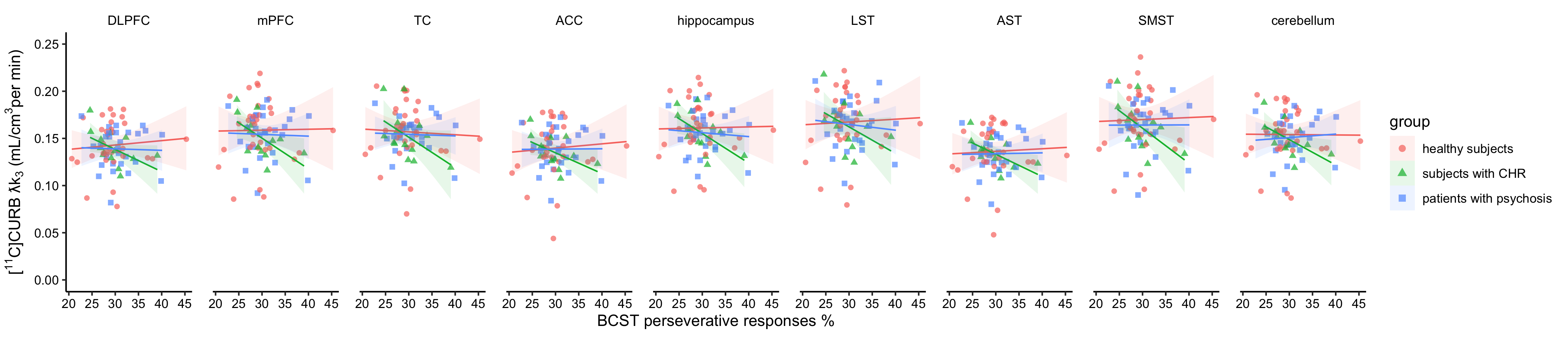
**


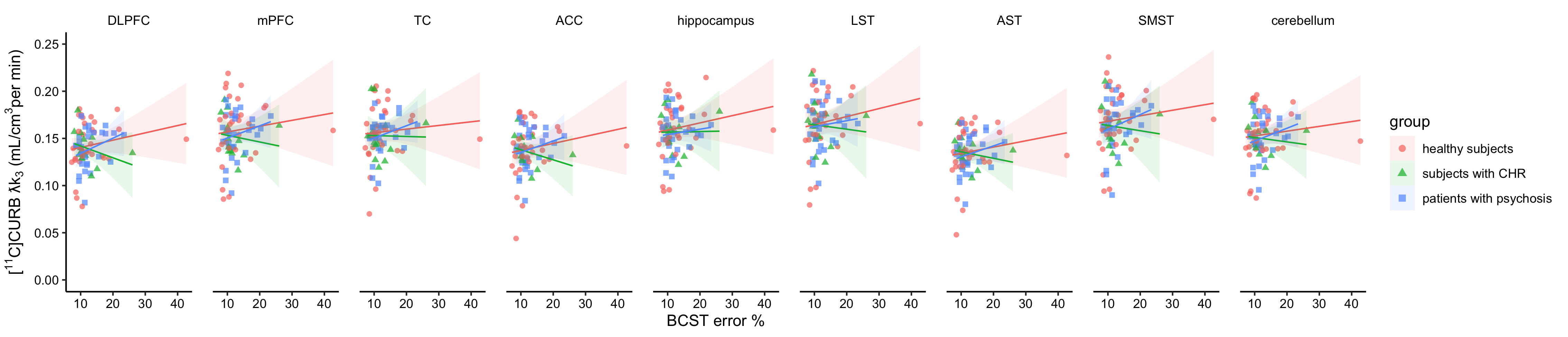


**Figure S2.** Correlations between BCST subtests (including BCST outlier) and [^11^C]CURB λk_3_ in healthy controls, clinical high risk for psychosis and patients with psychosis in different brain regions. DLPFC, dorsolateral prefrontal cortex; mPFC, medial prefrontal cortex; TC, temporal cortex; ACC, anterior cingulate cortex; LST, limbic striatum; AST, associative striatum; SMST, sensorimotor striatum.

### FAAH activity and cognitive performance between groups by regions of interest

**
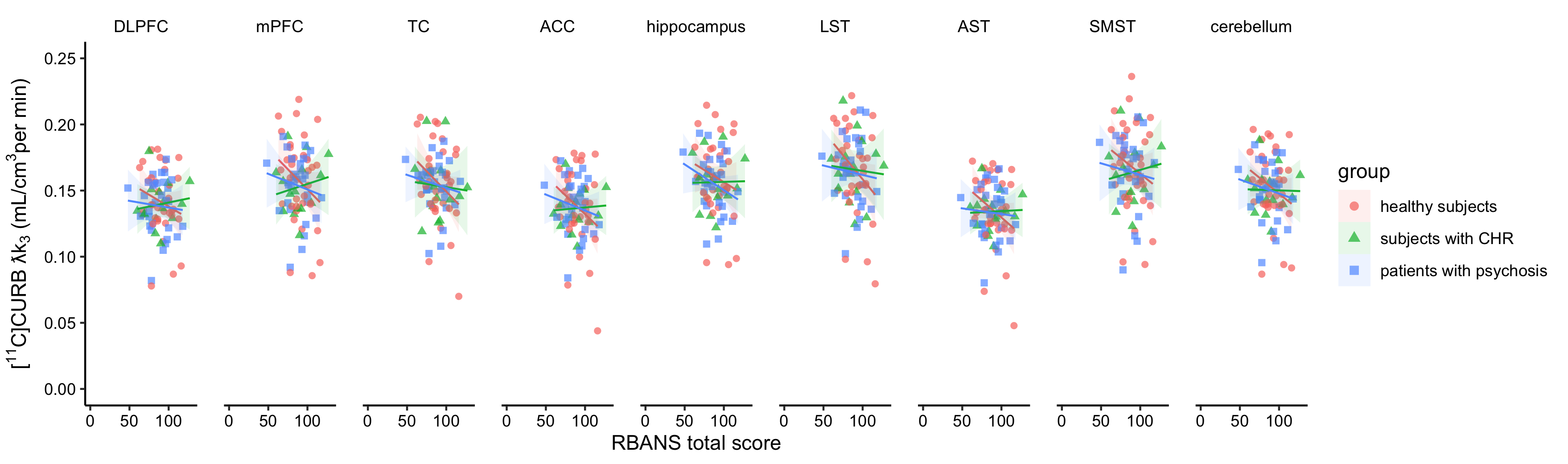
**


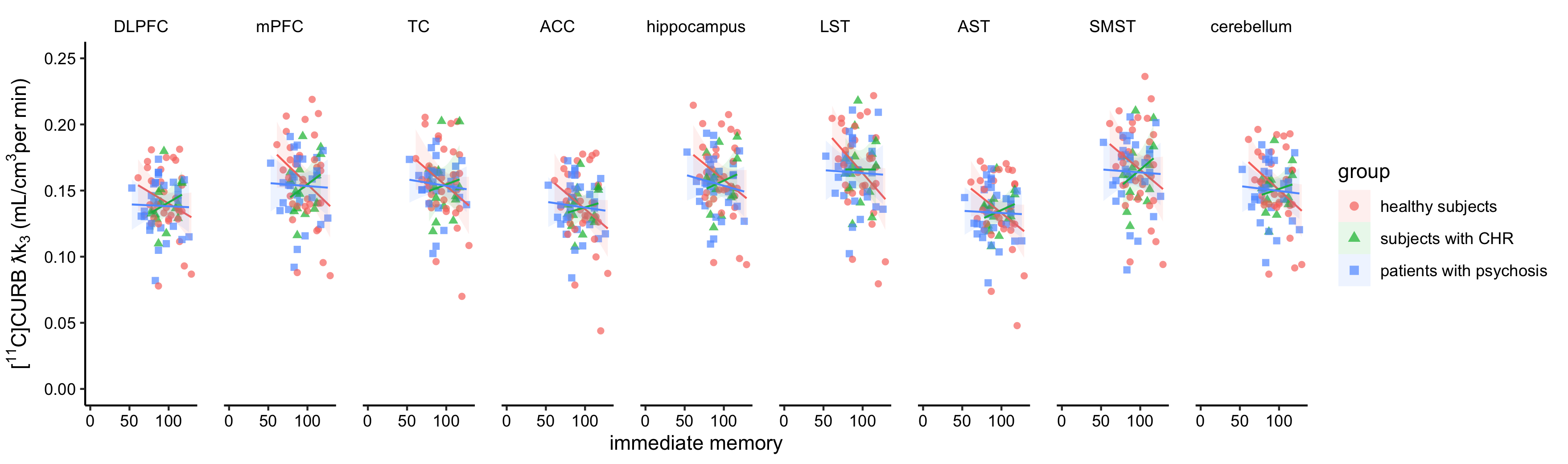


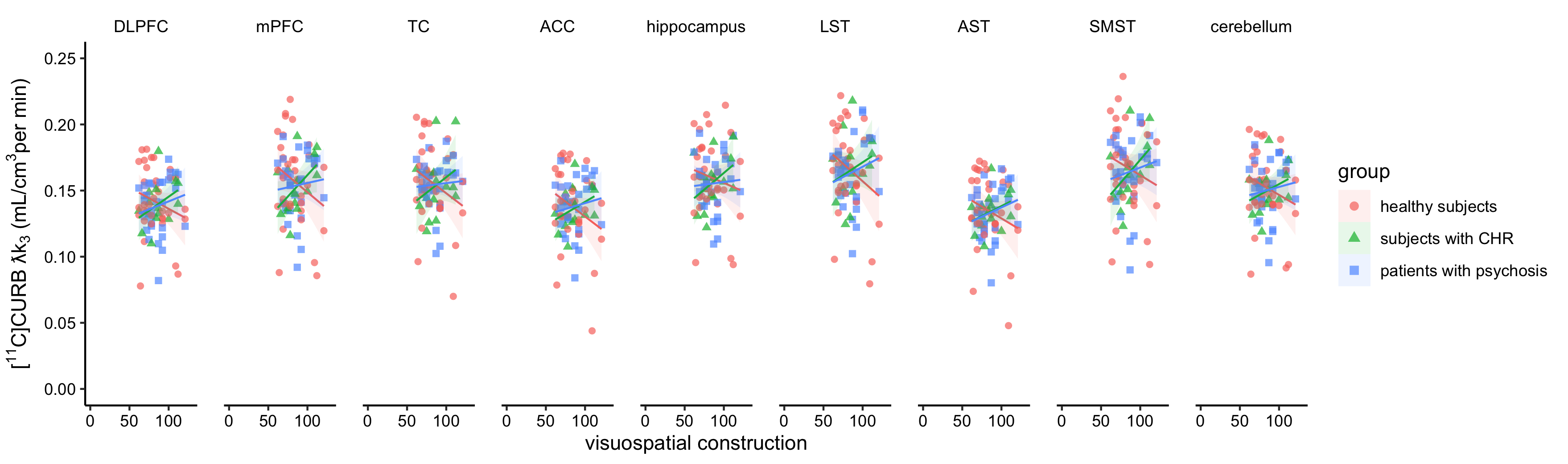


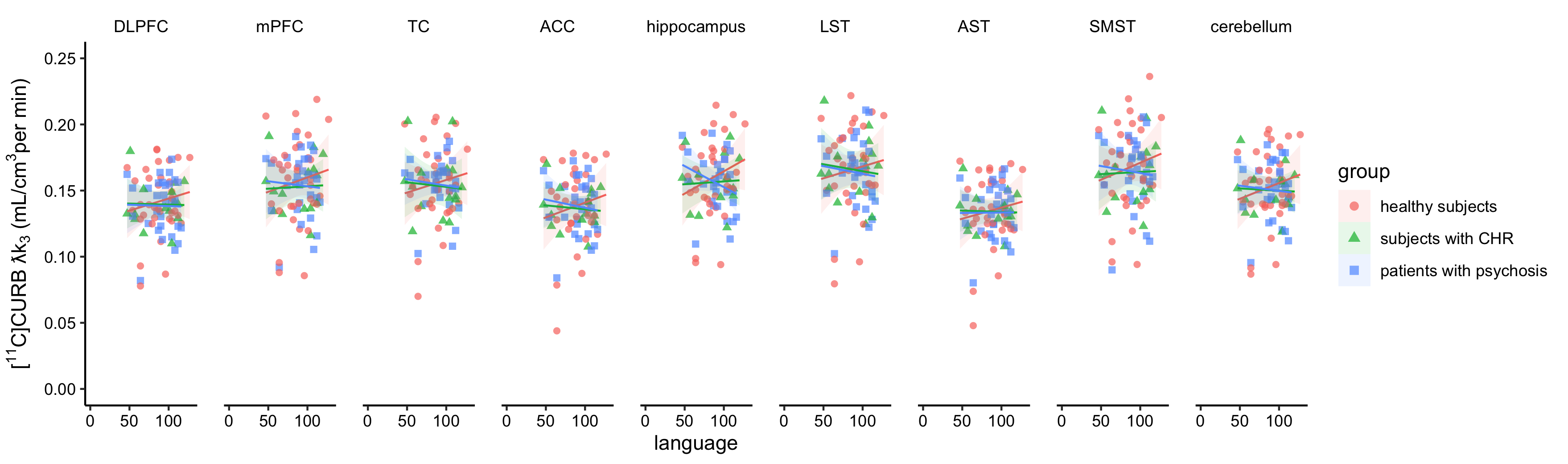


**
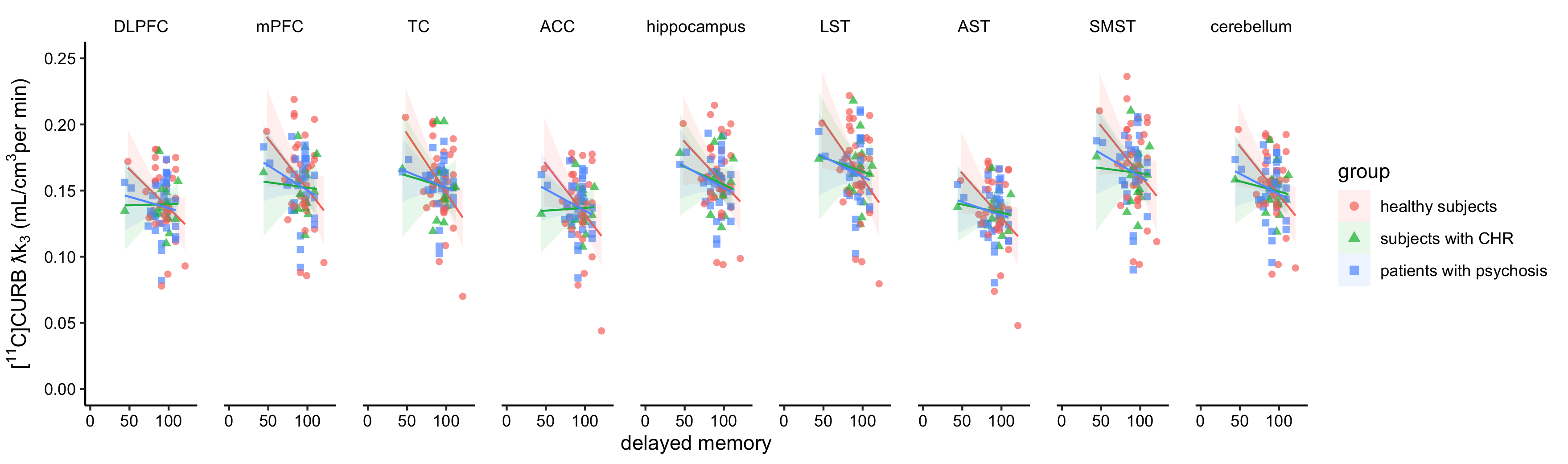

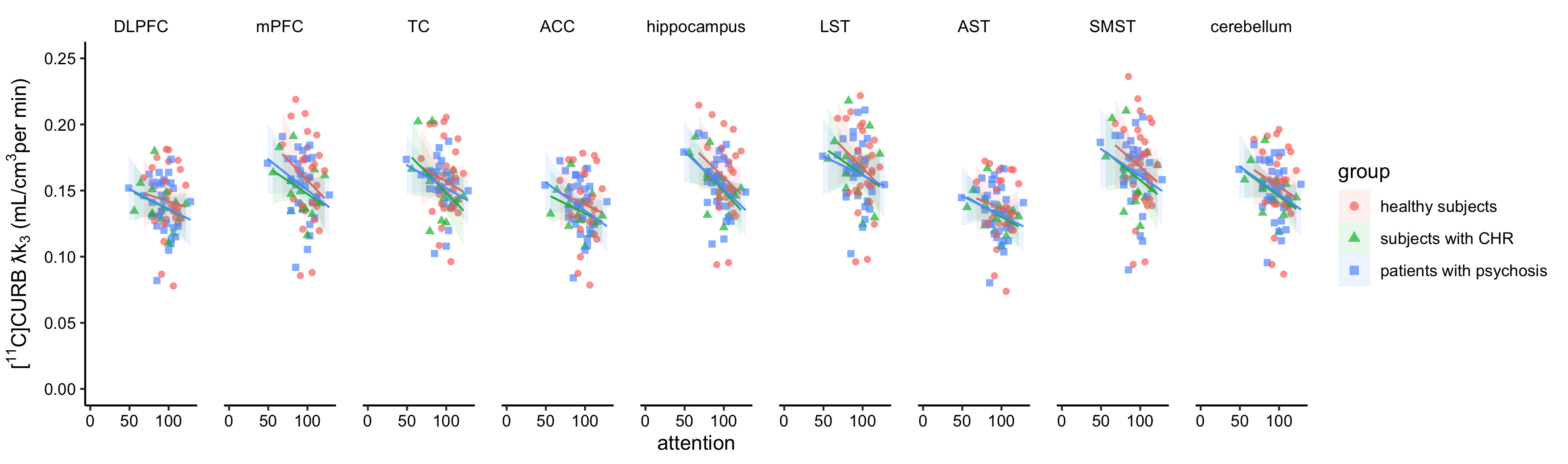
**

**
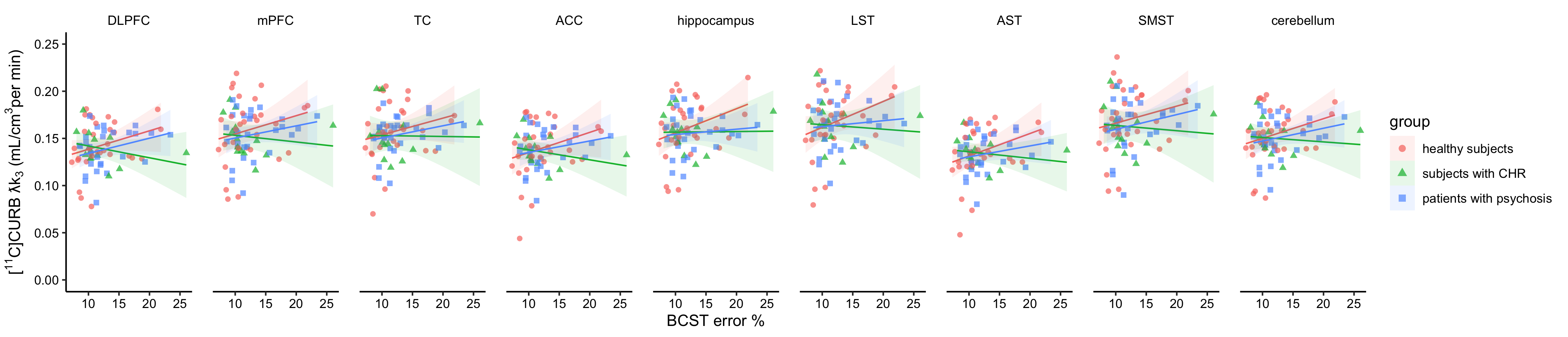
**

**
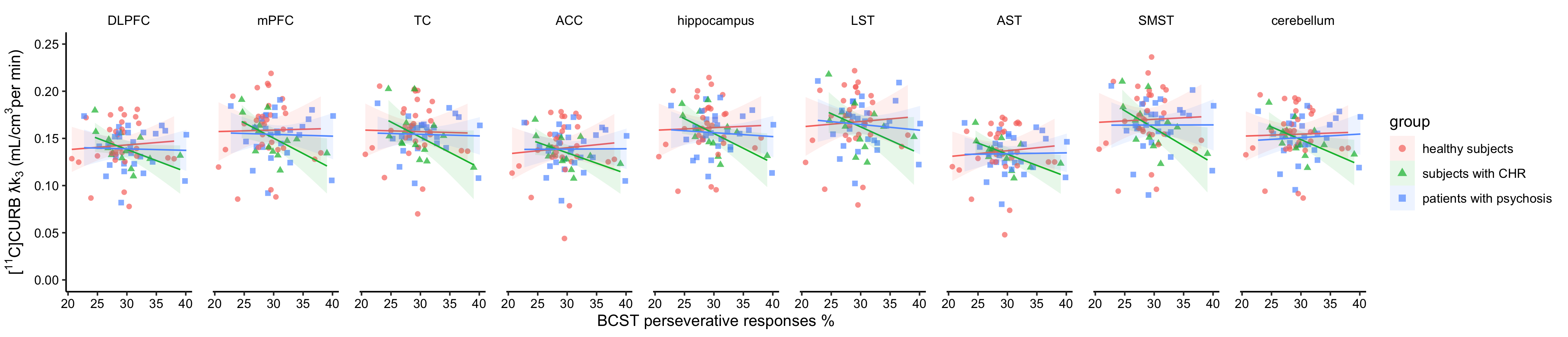
**

**Figure S3.**  Correlations between RBANS, BCST subtests and [^11^C]CURB λk_3_  in healthy controls, clinical high risk for psychosis and patients with psychosis in different brain regions. DLPFC, dorsolateral prefrontal cortex; mPFC, medial prefrontal cortex; TC, temporal cortex; ACC, anterior cingulate cortex; LST, limbic striatum; AST, associative striatum; SMST, sensorimotor striatum.

### Cannabis exposure and cognition

Correlations between past year exposure with RBANS total and each subscore individually, did not show any significance across groups. This was also true when tests were performed for each group separately (data not shown).

### FAAH activity and visuospatial construction between groups: Analysis after exclusion A/A genotype outlier.

Post hoc partial correlations excluding participants with A/A genotype did not significantly alter the outcomes. The analyses continued to show a negative association between FAAH activity and visuospatial construction in HC (r = -0.28, p <0.001), while the relationship was positive in CHR individuals (r = 0.34, p <0.001) and in FEP (r = 0.22, p < 0.001).


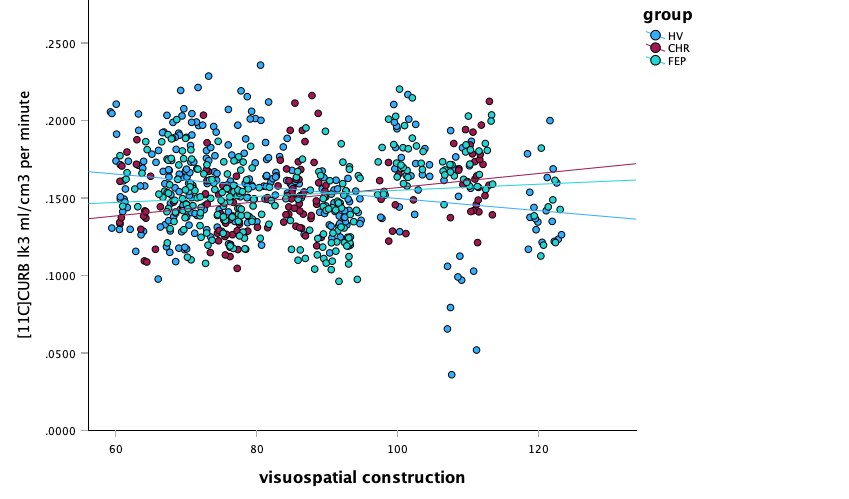


Fig S4. Correlations between RBANS visuospatial construction and [^11^C]CURB λk_3_, in healthy controls, clinical high risk for psychosis and patients with psychosis in different brain regions, excluding participants with A/A genotype. DLPFC, dorsolateral prefrontal cortex; mPFC, medial prefrontal cortex; TC, temporal cortex; ACC, anterior cingulate cortex; LST, limbic striatum; AST, associative striatum; SMST, sensorimotor striatum.
